# Infant-feeding Practices among HIV-infected Mothers in an HIV-treatment Programme

**DOI:** 10.3329/jhpn.v26i4.1890

**Published:** 2008-12

**Authors:** Wilson E. Sadoh, Ayebo E. Sadoh, Kayode A. Adeniran, Blessing I. Abhulimhen-Iyoha

**Affiliations:** 1 Department of Child Health; 2 Institute of Child Health, University of Benin/University of Benin Teaching Hospital, Benin City, Nigeria; 3 Department of Paediatrics, Federal Medical Centre Asaba, Asaba; 4 Department of Child Health, University of Benin Teaching Hospital, Benin City, Nigeria

**Keywords:** Antiretroviral therapy, Breastfeeding, Counselling, HIV, Infant-feeding practices, Infant food, PMTCT, Nigeria

## Abstract

The transmission of HIV via breastmilk has led to various recommendations for HIV-infected mothers. In this study, the feeding practices of HIV-infected mothers in the first six months of their infants’ lives were evaluated. In total, 103 consecutive mothers of children, aged 6-24 months, were evaluated for their feeding practices in the first six months of their infants’ lives. The mothers were recruited in two cohorts based on their entry (PMTCT cohort) or non-entry (non-PMTCT cohort) to an HIV MTCT-prevention programme. Information obtained included maternal age, socioeconomic class, and the educational level attained. All the babies in the non-PMTCT cohort were breastfed compared to none in the PMTCT cohort. Infant formula was inadequately prepared for 77.42% of babies in the non-PMTCT cohort compared to 18.64% in the PMTCT cohort. The mixed-feeding rate was high (70.45 %) in the non-PMTCT cohort. Over 70% of babies in both the cohorts were bottle-fed. Voluntary counselling and testing services in the healthcare system should be strengthened. All mothers should receive infant-feeding counselling, with exclusive breastfeeding being encouraged in those with unknown HIV status.

## INTRODUCTION

Exclusive breastfeeding is, reportedly, the ideal method for feeding infants in the first six months of life, as is recommended currently (1). The current recommendation for infants of HIV-infected mothers range from formula feeds with no breastfeeding at all (the ideal) to short periods of exclusive breastfeeding, followed by replacement feeding depending on the acceptability, feasibility, affordability, sustainability, and safety of the latter (2-4). These recommendations are predicated on the transmissibility of HIV via breastmilk. Mixed feeding is not recommended because of the associated higher transmission rate of HIV (5).

Several factors, such as non-affordability of alternative feeding, non-availability of potable water, and lack of information on appropriate feeding practices, especially in those who do not know their HIV status, may hamper the implementation of these recommendations in resource-poor settings.

It is with this background that the study was undertaken to evaluate the feeding practices of mothers with known HIV-positive status and women of unknown status (who were later confirmed to be HIV-positive) in the first six months of their infants’ lives in an HIV-treatment programme.

## MATERIALS AND METHODS

Consecutive mothers of children, aged 6-24 months, who were attending the paediatric HIV-treatment programme of the University of Benin Teaching Hospital (UBTH), Nigeria, were evaluated for their feeding practices in the first six months of their infants’ lives using an interviewer-administered questionnaire. Mothers were asked to recall their infant-feeding practices in the first six months of life. Verbal consent and information were obtained from the mothers. In three cases where the mothers were deceased, the father or the other caregiver was interviewed. Mothers who were diagnosed to be HIV-positive prenatally and who entered a Prevention of Mother to Child Transmission (PMTCT) programme were recruited in the PMTCT cohort, while those with unknown prenatal HIV status (but who were later found to be HIV-positive postnatally) and, therefore, did not enter a PMTCT programme, were recruited in the non-PMTCT cohort. Although mothers in the PMTCT cohort had infant-feeding counselling prenatally, those in the non-PMTCT cohort did not. The study was carried out during July 2005–December 2006.

Information on age and gender of infants and age, marital status, and level of education of mothers were obtained. Exclusive breastfeeding and mixed-feeding practices were assigned based on standard definitions (2-5). Information on the use of infant formula, which included adequacy of preparation and source of water for preparing formula, was also sought. The adequacy of the preparation of formula feeds was determined by comparing the number of scoops of formula added to a given amount of water (as prepared by the mother/ caregiver) following the recommendation of the manufacturer. The validity of information given was further evaluated by comparing the number of infant formula tins used per month with the standard recommendation (3,4). The formula preparation was inadequate when either or both means of evaluation was suboptimal. The introduction of, age at commencement and method of feeding cereal, adult food, water, and other fluids was ascertained.

The ELISA test was used for determining the HIV status of children aged over 18 months and DNA PCR in those aged less than 18 months. The socioeconomic class of the mothers was determined using the methods described by Olusanya *et al*. (6).

Data were analyzed using the SPSS software for Windows (version 10.0) (SPSS Inc., Chicago, IL). The differences in means were determined using *t*-test, while the association between proportions was tested by chi-square or Fisher's exact test or chi-square with Yate's correction when comparing small values. The level of statistical significance at 95% confidence interval was set at <0.05.

## RESULTS

Over the study period, 103 HIV-positive mothers and their infants were recruited. Of the mothers, 59 (57.28%) were recruited in the PMTCT cohort, and 44 (42.72%) were recruited in the non-PMTCT cohort. In the PMTCT cohort, 52 (88.14%) of the mothers had triple therapy of zidovudine, nevirapine, and lamivudine while seven (11.84%) mothers had single-dose nevirapine. Of the 103 infants, 42 (40.78%) were males. There was no significant difference in the sex distribution of the infants and maternal ages between the cohorts (Table 1).

**Table 1.** Sociodemographic characteristics of study population

Characteristics	PMTCT (n=59)	Non-PMTCT (n=44)	p value
Mean infant age (months)	11.85±4.88	16.98±6.40	0.0001
Maternal age (years)	30.69±3.85	30.55±4.82	0.8702
Gender (%)			
Male	20 (33.90)	22 (50.00)	0.149
Female	39 (66.10)	22 (50.00)	
Socioeconomic status (%)			
High SES	21 (35.59)	5 (11.36)	0.006
Middle SES	22 (37.29)	11 (25.00)	0.268
Low SES	16 (27.12)	28 (47.46)	0.0005
Maternal level of education (%)			
Primary	13 (22.03)	11 (25.00)	0.907
Secondary	28 (47.46)	27 (61.36)	0.230
Post-secondary	18 (30.51)	6 (13.64)	0.059

SES=Socioeconomic status

No baby in the PMTCT cohort was breastfed whereas all babies in the non-PMTCT cohort were breastfed. The rate of exclusive breastfeeding for infants in the non-PMTCT cohort was 15.91% (7 of 44 babies). The large majority (70.45%, n=31) of the babies in the non-PMTCT cohort had mixed feeding. All the babies in the PMTCT cohort had infant formula, of which 48 (81.36%) had adequately-prepared formula. Comparatively, 31(70.45%) babies in the non-PMTCT cohort received infant formula, with only seven (22.58%) receiving adequately-prepared formula. Most mothers—both in PMTCT cohort (81.35%, n=48) and non-PMTCT cohort (87.09%, n=27)—boiled water and then allowed it to be cool before using it to prepare the formula. The difference between the cohorts was not significant (p=0.367). The predominant source of water was untreated water from private bole holes.

There was no significant difference between the groups in the number of babies receiving cereals in the first six months and in the mean age when feeding cereal was commenced (Table 2). The majority (66.00%, n=33) of the babies who had cereal received pap fortified with milk or ground crayfish (Pap is a local gruel made from maize and contains mainly carbohydrate). Others had Custard (22%, n=11), Cerelac (8%, n=4), and Nutrend (4%, n=2). The mothers in the PMTCT cohort commenced feeding cereals because the babies (77.42%, n=24) became hungrier; mothers thought that babies were old enough (16.13%, n=5), and there was non-affordability (6.45%, n=2) of infant formula. In the non-PMTCT cohort, cereals were commenced because babies (66.67%, n=16) became hungrier; mothers felt that babies (16.67%, n=4) were old enough; maternal illnesses were the cause in three (12.50%) cases; and sorebreast was the cause in one (4.16%) case.

**Table 2.** Type of food/fluid fed to study population

Type of food/fluid	PMTCT (n=59)		Non-PMTCT (n=44)		p value	RR	95% CI
	No.	%	No.	%			
Breastmilk	0	0.00	44	100			
Infant formula	59	100	31	70.45	<0.0001		
Cereal	31	52.54	19	43.18	0.426	1.17	0.84-1.64
Adult food	8	13.56	7	15.91	0.783	0.92	0.55-1.52
Mixed feeds	0	00.00	31	70.45	<0.0001		
Water	45	76.27	25	56.82	0.059	1.46	0.96-2.21
Glucose water	3	5.08	6	13.63	0.166		
Soft drinks	1	1.69	1	2.27	1.000		

CI=Confidence interval; RR=Relative risk

Adult food was commenced in seven (15.91%) infants in the non-PMTCT cohort at a significantly lower mean age of 4.57±1.13 (range 3-5.9) months compared to a mean age of 5.43±0.52 (range 5-5.7) months in eight (13.56%) babies in the PMTCT cohort. Adult food consisted of mashed food during meals in the home. The reasons for giving adult food to babies in the PMTCT cohort were: babies becoming hungrier (25%, n=2,) and non-affordability of formula (75.00%, n=6). The reasons for giving adult food in the non-PMTCT cohort included four (57.14%) cases where the baby was thought to be old enough, two (28.57%) infants who became hungrier, and one (14.29%) infant due to the high cost of formula.

Infant formula or cereals were fed to 43 (72.88%) babies using feeding bottles while 16 (27.12%) were fed with cup and spoon in the PMTCT cohort. This was not significantly different from the non-PMTCT cohort where formula or cereals were fed to 22 (70.97%) infants using feeding bottles, and nine (29.03%) were fed with cup and spoon (p=1.000).

The large majority (74.58%, n=44) of mothers in the PMTCT cohort received antenatal care at the UBTH while 15 (14.56 %) had their antenatal care in other health facilities. In the non-PMTCT cohort, only one mother had antenatal care at the UBTH while 41 (93.18%) received antenatal care in other health facilities, and two had no antenatal care. Of 44 infants in the non-PMTCT cohort, 31 were HIV-positive, giving a transmission rate of 70.45%. The rate was significantly higher than 8.47% for those who were in the PMTCT cohort (p=0.0001; relative risk [RR]=5.803, CI 2.55-13.200).

## DISCUSSION

The study revealed the differences in the feeding practices between mothers based on entry or non-entry into the MTCT intervention programme for their babies. All the mothers in the PMTCT cohort chose not to breastfeed their babies having received infant-feeding counselling prenatally. Willingness of mothers to choose this option, if they were HIV-positive, has been previously reported (7). In contrast, all the mothers in the non-PMTCT cohort breastfed their babies, and the large majority (70.45%) of their babies had mixed feeding. These findings are in agreement with those of a Zimbabwean study where incorrect feeding practices were observed among HIV-positive mothers who were unaware of their HIV status (8). That the mothers in the non-PMTCT cohort could have benefited from infant-feeding counselling is shown by their tendency to stop breastfeeding once they knew their HIV status. This tendency may explain the low rate of exclusive breastfeeding observed in them compared to an earlier study in Benin City (9).

More mothers who received infant-feeding counselling compared to those who did not prepared infant formula adequately. However, about a fifth of these mothers did not prepare their infants’ formula adequately, suggesting the possibility of other contributory factors, such as non-affordability/non-availability of formula. This may be buttressed by the citing of non-affordability of formula feed by mothers in the same cohort as the reason for commencing either cereal or adult diet. The inability of mothers to provide adequate formula feed to their babies has been previously reported (10).

In keeping with the results of the previous studies, the majority (66%) of the mothers in this study gave pap enriched with milk or ground crayfish (9,11). Pap is indigenous and, hence, was the predominant source of cereal. All mothers who gave pap enriched it to improve its nutritional value—an improvement on the less than half of mothers who fortified pap in an earlier study in the same locality (9).

Most mothers in both cohorts fed their babies with bottles, a practice that has been associated with a higher frequency of diarrhoeal diseases compared to feeding with cup and spoon (2,4). It is possible that this method of feeding was not emphasized during infant-feeding counselling.

The effect of no breastfeeding in reducing the rate of transmission of HIV from the mother to the child in the PMTCT cohort was evident in this study and corroborated the findings of the previous studies (5,12). The use of antiretroviral drugs in mothers and babies also contributed. The transmission rate in the PMTCT cohort was, however, higher compared to other studies where more than one drug was used (13,14). This may have been due to the fact that the mothers were exposed to a different antiretroviral regimen, including single-dose nevirapine for mother and baby. The high rate of transmission of HIV in the non-PMTCT cohort could be partly explained by the multiple effects of breastfeeding, high mixed-feeding rate, and lack of maternal and child exposure to antiretroviral drugs. Besides, more mothers from this cohort were from low socioeconomic status and were poorly educated. This could have negatively affected their willingness to get tested.

Although most mothers in the study attended antenatal care at a government hospital or a private clinic, only those who sought care in the study centre where the PMTCT facility is located had the benefit of interventions.

One limitation of the study was that there was no standardization of ages of the children when the mothers were interviewed. This bias in the recall ability of the mothers might have influenced some responses.

Based on the findings of the study, it is recommended that all health facilities offering antenatal care services should provide voluntary counselling and testing services so that HIV-positive pregnant women can enter a mother-to-the-child transmission intervention programme. All mothers should receive infant-feeding counselling, with exclusive breastfeeding being encouraged in those with unknown HIV status. The need to closely evaluate the ability of HIV-positive mothers to provide replacement feeding in terms of its acceptability, feasibility, affordability, sustainability, and safety in line with the recommendations of the World Health Organization, as part of infant-feeding counselling, is imperative (15).
